# Development and piloting of a plan for integrating mental health in primary care in Sehore district, Madhya Pradesh, India

**DOI:** 10.1192/bjp.bp.114.153700

**Published:** 2016-01

**Authors:** Rahul Shidhaye, Sanjay Shrivastava, Vaibhav Murhar, Sandesh Samudre, Shalini Ahuja, Rohit Ramaswamy, Vikram Patel

**Affiliations:** **Rahul Shidhaye**, MD(Psych), MHS, Public Health Foundation of India, Bhopal, Madhya Pradesh, India, and CAPHRI School for Public Health and Primary Care, Maastricht University, The Netherlands; **Sanjay Shrivastava**, MBBS, **Vaibhav Murhar**, MA(Psychol), PRIME project (India), Sangath, India; **Sandesh Samudre**, BAMS, MPH, **Shalini Ahuja**, MHMPP, BPT, Public Health Foundation of India; **Rohit Ramaswamy**, PhD, MPH, Grad.Dipl.(Bios), Public Health Leadership and Maternal and Child Health, University of North Carolina, Chapel Hill, North Carolina, USA and Adjunct Faculty, Public Health Foundation of India; **Vikram Patel**, MRCPsych, PhD, FMedSci, London School of Hygiene & Tropical Medicine, London, UK, Public Health Foundation of India and Sangath, India

## Abstract

**Background**

The large treatment gap for mental disorders in India underlines the need for integration of mental health in primary care.

**Aims**

To operationalise the delivery of the World Health Organization Mental Health Gap Action Plan interventions for priority mental disorders and to design an integrated mental healthcare plan (MHCP) comprising packages of care for primary healthcare in one district.

**Method**

Mixed methods were used including theory of change workshops, qualitative research to develop the MHCP and piloting of specific packages of care in a single facility.

**Results**

The MHCP comprises three enabling packages: programme management, capacity building and community mobilisation; and four service delivery packages: awareness for mental disorders, identification, treatment and recovery. Challenges were encountered in training primary care workers to improve identification and treatment.

**Conclusions**

There are a number of challenges to integrating mental health into primary care, which can be addressed through the injection of new resources and collaborative care models.

The contribution of mental disorders to the overall burden of disease in India in 2010 was estimated to be 5.6%.^1^ Although the World Health Organization (WHO) Mental Health Gap Action Plan (mhGAP) guidelines testify to the cost-effectiveness of a wide range of drug, psychological and social interventions that can transform the lives of people affected by mental disorders,^2^ it is estimated that in India only about 10% of people with mental disorders are receiving evidence-based interventions.^3^ India was one of the first of the low- and middle-income countries (LIMC) to start a national mental health programme in 1982 with the aim of extending community-based mental healthcare through the existing primary healthcare system.^4^ The district mental health programme (DMHP) was conceptualised during 1984–90 and currently is in operation in 125 districts (out of the total 626 districts).^4^ However, evaluations of the DMHP indicate that it is, to a large extent, ineffective in practice.^3^ Some of the reasons for this unsatisfactory state of affairs are: the top–down, ‘one size fit all’ approach to service delivery that cannot accommodate diverse local realities, poor governance, managerial incompetence and unrealistic expectations from low-paid or poorly motivated primary healthcare personnel.^4^ Despite these challenges, several recent developments at the national level offer a conducive policy environment for strengthening the mental health system in India and improving the delivery of mental health services. The draft Mental Health Care Bill, which was tabled in the Indian Parliament in August 2013, enshrines access to community mental healthcare as a human right.^5^ The Ministry constituted a mental health policy group in April 2011 to prepare a national mental health policy and plan. Based on the document reviews, consultations with key stakeholders and field visits, the mental health policy group proposed substantial changes to the design of the DMHP for the twelfth 5-year plan period (2012–2017).^6^ Thus, there is a robust policy context for designing and implementing a district-level MHCP that will help operationalise the rejuvenated DMHP. However, little is known about the mechanisms for implementing integrated MHCPs in local district settings in India. Under the auspices of the PRogramme for Improving Mental health carE (PRIME),^7^ we sought to achieve this goal in the district of Sehore in the central state of Madhya Pradesh. In this paper we describe the process of development of the MHCP, its framework and content, and experiences from the pilot implementation.

## Method

### Setting

The research was implemented through a partnership between Sangath (a non-governmental organisation (NGO) working in the sector of public mental health), the Public Health Foundation of India and the Ministry of Health, Government of Madhya Pradesh. Madhya Pradesh is situated in the central part of India and has a population of 72.5 million, which accounts for 6% of the total population of India. Madhya Pradesh was selected because it was a priority state for UK aid on the grounds of its poor general health indicators. Sehore district, which lies in the south-west direction of the state capital, Bhopal, has a population of 1.3 million that is predominantly rural (81%) and covers an area of 6578 km^2^ (see online Fig. DS1).

Sehore district was selected because it is one of the districts where the DMHP has already been implemented and therefore the infrastructure to develop, implement and scale up the MHCP was already in place. UK aid had no role in either the selection of the state (i.e. Madhya Pradesh) or the district (i.e. Sehore) and it was entirely based on the decision of the PRIME team in consultation with the Ministry of Health.

### Overview of methodology

**Fig. 1:**
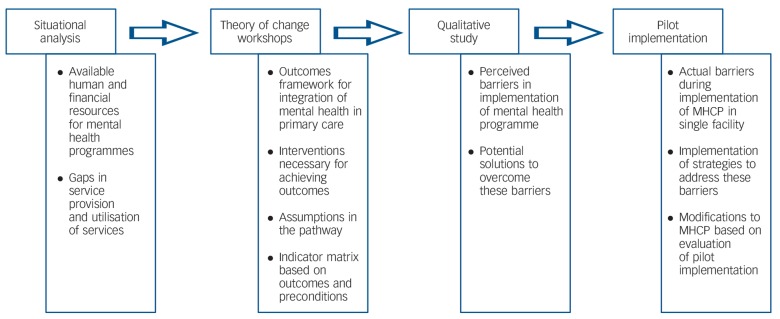
Process of development of the mental healthcare plan (MHCP).

The MHCP for Sehore district was developed during the period August 2011 to March 2014. This approach broadly followed the guidance for developing and implementing complex interventions provided by the Medical Research Council.^8^ This recommends synthesising the evidence on effective treatments for the target conditions, formative research that includes modelling the processes through which care will be delivered and pilot testing them before rolling out the intervention and a definitive evaluation of the final implementation is carried out.^9^ The recent evidence base in global mental health such as WHO mhGAP treatment guidelines^10^ and the *PLoS Medicine* series on global mental health^11^ served as the foundation and a starting point for creation of draft packages of care for priority mental disorders constituting the MHCP. Depression, psychosis and alcohol use disorders were selected as the three priority mental disorders as these are the three leading mental health causes of the burden of disease^12^ and there is a growing body of evidence testifying to both the efficacy of specific treatments for these disorders and their cost-effectiveness.^11^ It was also decided that the depression package would include interventions for maternal depression as it is the second leading cause of disease burden in women worldwide, following infections and parasitic diseases.^10^ We then carried out formative research to understand the local context of Sehore district and model the mental health packages that could be delivered in this context. This was followed by pilot testing specific packages of the MHCP in a single facility to evaluate their acceptability and impact, and making adaptations to the plan based on learning from the pilot. This overall process is shown in Fig. 1.

### Description of specific methods

#### Situational analysis

The situational analysis comprised a document review of Sehore district's DMHP and other health-related documents to summarise existing knowledge about mental health service delivery in the district. A cross-country situational analysis tool developed by the PRIME team served as the basis for conducting the document review. The details of the situational analysis are described elsewhere.^11^ The situational analysis primarily helped us to identify the available human and financial resources, gaps in mental health service provision in Sehore district and the issues related to mental health service utilisation such as the role of stigma and traditional healers in help-seeking behaviour.

#### Theory of change (ToC) workshops

ToC is an outcomes-based approach that applies critical thinking to the design, implementation and evaluation of programmes intended to support change in their contexts.^12^ These kinds of approaches are recommended to obtain a theoretical understanding of how complex interventions cause change. We used this approach to design the MHCP and obtain the opinion of key stakeholders about the clinical and health system outcomes that need to be realised in order to develop and implement a successful MHCP. A series of ToC workshops were conducted to build stakeholder consensus around essential packages of the MHCP for the three priority disorders. Two workshops were conducted, the first in January 2012 and the second in April 2012 involving 17 participants (state- and district-level planners, health service providers, paramedical and front-line workers). The details of this are described elsewhere.^13^ The output of these ToC workshops is a ToC map that describes the chain of activities that need to be completed to deliver mental health services. Based on this ToC map, four key processes were identified: awareness, detection, treatment and recovery. The discussions in the ToC workshops also highlighted that effective service provision and utilisation can only be achieved in a supportive and enabling environment provided by the healthcare organisation. The framework provided by ToC was the basis for the design of the first draft of the MHCP, which was broadly divided into service delivery packages and enabling packages. Each of these packages was further divided into smaller components, and for each of the components a process map was developed that depicted the actual flow or sequence of events for activities or processes in that particular component. This exercise clearly defined where the process under study starts (input) and ends (final impact).

#### Qualitative study

The qualitative study consisted of in-depth interviews and focus-group discussions with stakeholders and was conducted in the month of March–April 2012. The sampling of respondents for in-depth interviews was purposive to ensure that the perspectives of all stakeholders in mental health service use and provision was obtained. A total of 11 in-depth interviews were conducted, with 4 state-level policy makers, 3 specialists working in the Department of Health Services and 4 general health service providers and health managers.

Four focus-group discussions were conducted in two blocks (subdistricts with a population of around 300 000) i.e. Ashta and Budhni subdistricts. Out of these, two focus-group discussions were conducted with paramedical staff in primary healthcare facilities (multipurpose workers, pharmacist, auxiliary nurse midwife); front-line workers (who were accredited social health activists, volunteers with roles similar to a community health worker); Anganwadi workers (community health workers mainly focusing on maternal and child health components based in the community) and with community members (both male and female members of the village). Eight participants participated in each of these four focus-group discussions. A framework analysis approach was used to analyse the qualitative data. An *a priori* coding framework with a set of high-level themes was developed: (a) availability of mental health services; (b) quality of services provided; (c) demand for services focusing on awareness and stigma; (d) rehabilitation facilities; (e) capacity-building issues; (f) monitoring and information systems; (g) drug supply; (h) health system requirements for mental health service delivery; and (i) mental health policy. Other lower-order themes were inductively derived from the data. NVivo9 qualitative data analysis software was used to store and to code the data using the developed coding framework.

#### Pilot implementation

One of three community health centres in Sehore district (Bilkisgunj) was selected for pilot implementation of the MHCP. A community health centre is the equivalent of a subdistrict hospital and caters for a population of around 150 000 in Madhya Pradesh. The identified facility is situated in a town but mostly serves the rural population and on average there are 1600 outpatients (including adults and children) per month (in 2013). Pilot implementation was carried out during the period of July 2013 to March 2014. During this 9-month period enabling packages were piloted, related to capacity building, procurement and supply chain management, health management information system (HMIS) and service delivery packages related to detection and treatment of priority mental disorders. Evaluation of pilot implementation was carried out by collecting quantitative data related to process indicators such as number of patients diagnosed by the primary care physicians, number of patients receiving care in specialist clinics and their sociodemographic and clinical characteristics, direct observation of the care pathways and processes and a qualitative study to explore the acceptability and feasibility of the packages of care through in-depth interviews with the DMHP team (*n* = 3), medical officers (*n* = 3), front-line workers (*n* = 6), patients (*n* = 6) and carers (*n* = 4). In addition, one focus-group discussion was conducted with patients and carers (*n* = 8).

### Data analysis

The data obtained from situational analysis, ToC workshops and the qualitative study were triangulated to define packages of care and develop a detailed MHCP. Barriers to implementation of the MHCP were identified during the qualitative study and were further evaluated during the pilot implementation. Finally, the data from evaluation of the pilot helped us to identify potential solutions to address these barriers and modify the MHCP for the full roll-out in the implementation area. Ethical approval was obtained from the Institutional Review Board of Sangath and the programme was approved by the Indian Council for Medical Research.

## Results

### Packages of care in the MHCP

The MHCP consists of service delivery and enabling packages. Each package can be further broken down into components and for each, a number of steps for their implementation are identified. The enabling packages consist of cross-cutting interventions that will ensure the smooth implementation of core mental health service delivery packages. There are three enabling packages: programme management, capacity building and community mobilisation. The key implementation steps human resources required in enabling packages are described in Table 1.

**Table 1:** Mental healthcare plan: enabling packages^a^

Package and component	Implementation steps	Human resource^a^
Programme management		
Procurement and supply chain management	Timely approval of the requests from facilities	District-level administration/chief medical and health officer
	Request for procurement of psychotropic drugs generated from community health centres/district hospital Maintenance of buffer stock of psychotropic drugs	Facility administration/in-charge medical officer and *PRIME district coordinator*
Mental health information systems	Compilation of data on monthly basis done at the district level	District-level administration/health management information system officer and *PRIME district coordinator*
	Feedback sent to the facilities (community health centres) based on the indicators	
	Information on key mental health service delivery indicators sent to the district on weekly basis	Facility administration/in-charge medical officer
Human resource management	Review of current human resource for mental health service delivery	District-level administration/chief medical and health officer
	Engagement with potential human resource (existing/new) for mental health service delivery	
	Administrative supervision and performance assessment of human resource	
Financial management	Set up of financial management standards as per government guidelines	District-level administration/chief medical and health officer
	Directives for prompt utilisation of funds and to ensure reporting	
	Provide feedback and corrective action Ensure timely utilisation and reporting	Facility administration/in-charge medical officer

Capacity building		
Curriculum development	Design and contextualisation of training materials	*PRIME intervention coordinator*
Training and supervision	Conduction of training sessions for medical officers and front-line workers Supportive supervision sessions for medical officers and front-line workers	District mental health programme team and *PRIME intervention coordinator*

Community mobilisation		
Community engagement	Engage with community members and key stakeholders Formulate and ensure smooth functioning of user groups	*PRIME intervention coordinator and district coordinator*
Partnership building and resource mobilisation	Identification and mapping of governmental/ non-governmental organisations interested in mental health programmes Establishment of district mental health consortium	*PRIME intervention coordinator and district coordinator*

PRIME, PRogramme for Improving Mental health carE.

a. Plain text indicates individuals who were responsible for implementing these packages during the design of the mental healthcare plan, whereas text in italics indicates individuals who need to play an important role in implementation of these packages post-pilot implementation.

The four service delivery packages are awareness for mental disorders, identification, treatment and recovery. The core service delivery packages are related to the delivery of mental health services for the three priority disorders: depression, psychosis and alcohol use disorders. The key implementation steps and the human resources required for the core service delivery packages are described in Table 2.

**Table 2:** Mental healthcare plan service delivery packages^a^

Awareness	Detection	Treatment	Recovery	Human resource
Specialist/district hospital				
Conduct small group meetings with individuals attending the hospital Screen audiovisual interventions/films in the hospital Display of posters in a designated space/ corner in the hospital	History-taking, assessment and clinical diagnosis of priority disorders of individuals coming to the hospital/referred from facility	Prescribe antidepressants and psychosocial interventions to individuals with depression as per mhGAP guidelines Management of psychosis as per mhGAP guidelines Management of alcohol dependence Provide psychoeducation to families of individuals with depression, alcohol use disorders and psychosis Facilitate referral of individuals with depression, alcohol use disorders and psychosis to higher centres for further management	Link individuals with depression, alcohol use disorders and psychosis to community health centres (if required) for further follow-up and community-based rehabilitation	District mental health team (psychiatrist and psychologist) *Mental health case manager*

Community health centres/ subdistrict hospital				
Conduct small group meetings with individuals attending the facility Screen audiovisual interventions/films in the facility Display of posters in a designated space/corner in the facility	History-taking and assessment of priority disorders based on mhGAP guidelines, of individuals coming to the facility/referred by front-line workers	Prescribe antidepressants and psychosocial interventions to individuals with mild to moderate depression as per mhGAP guidelines Manage acute episode of psychosis as per mhGAP guidelines Provide brief interventions to individuals with alcohol use disorders as per mhGAP guidelines Provide psychoeducation to families of individuals with depression, alcohol use disorders and psychosis Facilitate referral of individuals with depression, alcohol use disorders and psychosis to district level for further management	Provide follow-up care including prescription of psychotropic medications and psychosocial interventions to individuals referred from the district hospital after initial consultation Link individuals with depression, alcohol use disorders and psychosis to front-line workers for further follow-up and community-based rehabilitation	Medical officers and paramedical workers *Mental health case manager*

Community				
Conduct small group meetings with community members Screen audiovisual interventions/films in villages Display of posters at community meeting places such as village government offices (Gram Panchayat) and village health and nutritio centre (Anganwadi)	Establish contact, interact and assess individuals based on symptoms/behavioural presentations	Provide mental health first aid with emphasis on self-care strategies especially for depression Provide psychoeducation to families of individuals with psychosis and encourage referral Provide brief interventions to individuals with alcohol use disorders Conduct risk assessment for self-harm Facilitate referral to facility by accompanying the individual if possible	Regular follow-up visits and advice on adherence support to individuals who have been prescribed drugs in the facility/advised psychosocial interventions Link individuals with psychosis (clinically stabilised) to rehabilitation agencies in the community Link individuals with alcohol use disorders to existing self-help groups such as Alcoholic Anonymous	Front-line workers *Mental health case manager*

mhGAP, World Health Organization Mental Health Gap Action Programme.

a. Plain text indicates individuals who were responsible for implementing these packages during the design of the mental healthcare plan, whereas text in italics indicates individuals who need to play an important role in implementation of these packages in the modified MHCP post-pilot implementation.

The enabling and service delivery packages intersect in a dynamic manner. For example, one of the major enabling packages is capacity building. The objective of the capacity-building package is to ensure that the medical officers, paramedical staff and front-line workers are trained in evidence-based interventions and continuous supportive supervision is provided to maintain and enhance the skills and competencies acquired during initial training. Once the enabling capacity-building package is functional it will ensure that the two service delivery packages related to identification and treatment can start functioning. These service delivery packages need to be complimented by the simultaneously functioning programme management package, which comprises four components: human resource management, financial resource management, supply chain management of essential psychotropic drugs and a well-functioning HMIS. Demand for mental health services will be improved by an awareness package (one of the service delivery packages) and this package will be supported by another enabling package related to community mobilisation. Engagement with the general community members and mobilising users, carers and other community members to advocate for rights-based delivery of mental health services is the objective of this enabling package. Thus, the enabling packages act as a foundation for service delivery packages of the MHCP.

### The piloting of the MHCP

The pilot implementation had three phases: capacity building and assessment of performance, followed by reviewing of barriers for implementation and strategies to address these, and finally the implementation of new strategies and their evaluation. The first two phases lasted approximately 4 months and the final phase was conducted over 5 months.

The capacity-building package was piloted in July 2013. Training programmes were jointly conducted by the DMHP and PRIME teams for medical officers to train them for delivery of the mental health packages identified in the MHCP. The WHO mhGAP training material served as the basis for this course and was conducted over 2 days.^14^ A separate 2-day training programme was carried out by the PRIME team for 20 front-line works and 15 paramedical staff. The training focused on an introduction to mental health, how to detect these disorders in the community setting, where to refer and how to provide mental health first aid. Following the training, medical officers were followed up weekly in a face-to-face meeting with the PRIME team. Data related to number of patients identified, treatment initiated and patients referred (if required) was gathered. In the first month of follow-up, only seven patients were detected and three patients were referred to the district hospital by seven medical officers. Out of the seven patients, five had depression and one each had psychosis and alcohol use disorders. Basic psychoeducation was provided to four patients with depression (the remaining being directly referred to the district hospital). No pharmacological treatment was provided to any of these patients by the medical officers as none of the psychotropic medications on the essential drug list were available in the facility. No formal reporting to the district health system was done as reporting on mental health indicators was not part of the existing HMIS. The front-line workers were also followed-up on a weekly basis in the first month following the training; none of them had identified or referred any patient to the facility. There was no initiation of any mental health first aid at the community level. In summary, the training of medical officers and front-line workers resulted in a very small number of patients being detected and started with treatment for priority mental disorders. There were no interventions taking place at the community level, no linkage was established between the DMHP team and the facility team, and there was no availability of psychotropic medications, and no monitoring and HMIS was functional.

A review meeting with the DMHP team and facility team (medical officers, paramedical workers and front-line workers) was conducted to identify the barriers to the implementation of the MHCP and the strategies to address these (Table 3). The focus of these strategies was to mobilise the human resources at the facility level and engage them in mental health service delivery. The PRIME team members were given the task of facilitation of community-based activities by establishing contacts with front-line workers (accredited social health activists workers) and their supervisors. The PRIME district programme coordinator played a central role in establishing liaison with district and state administration to obtain the required directives mentioned in Table 3. Finally, the DMHP team was requested to conduct specialist clinics in the facility to address the needs of patients mobilised by the community interventions.

**Table 3:** Strategies to address barriers during the first phase of pilot implementation

Barriers in implementation	Strategies to overcome these barriers
No identification of patients with priority disorders at the community level by front-line workers and non-provision of mental health first aid	Facilitation by the PRIME team members of community-based activities by establishing contacts with front-line workers (accredited social health activists workers) and their supervisors

Low identification and treatment initiation for priority mental disorders by facility medical officers Non-establishment of linkage between facility medical officers and specialist at the district level	Specialist clinics by district mental health programme team (psychiatrist and psychologist) to be organised in the facility to provide clinical services. In addition to the clinics they will also interact, train and supervise the work of medical officers and paramedical workers

Non-availability of psychotropic drugs in the facility	Clear directives from state- and district-level administration to be obtained for supply of essential psychotropic drugs. The PRIME team members will also work with medical officers to strengthen the process bottom-up

Non-reporting of mental health indicators	Clear directives from state- and district-level administration to be obtained for reporting on key performance indicators related to mental health service delivery such as number of patients with priority mental disorders identified. The PRIME team members will also work with medical officers to strengthen the process bottom-up

PRIME, PRogramme for Improving Mental health carE.

The new strategies were implemented from November 2013 to March 2014. Community-based identification, provision of mental health first aid and referral of patients to a facility was primarily done by front-line workers. They established direct contact with families in which individuals were suspected to have priority mental disorders. Identification of individuals was based on a symptom profile for the priority mental disorders mentioned in mhGAP guidelines. Those who were found to have mental disorders were provided mental health first aid. The PRIME team members established a strong linkage with the supervisors of the front-line workers and facilitated the referral of the identified individuals to the facility for outreach clinics where they were provided with pharmacological treatment and psychosocial interventions. During December 2013 to March 2014, six specialist clinics were conducted in the facility on a fortnightly basis and 214 patients were seen. Almost two-thirds of the patients presented either with medically unexplained symptoms (59%) or depression (9%). Alcohol use disorders was diagnosed in 12% of patients, psychosis in 3% and epilepsy in 4% of the patients. Children and adolescents with intellectual disability also accessed care in these clinics (12%). Only six mothers with depression were detected and received the psychosocial interventions. Almost all the patients were prescribed medication by the psychiatrist and, from the second clinic onwards, patients also received basic psychosocial counselling, which focused on problem-solving, self-help strategies and advice about drug adherence by the PRIME team members and which was supervised by a DMHP psychologist. Paramedical workers in the facility should have provided the psychosocial counselling but as they never participated in the specialist clinics this task had to be undertaken by the PRIME team members. The psychiatrist working with the DMHP stated that specialist clinics provide an excellent opportunity to integrate mental health in primary care. Awareness about mental disorders and the need to treat them increased among the staff of the facility as observed through the number of people accessing services in these specialist clinics.

Since the implementation of new strategies, it was observed that the involvement of facility staff and their ownership of the programme improved as reflected by the provision of a space for specialist clinics and improved supply of psychotropic drugs. Psychotropic medication prescribed to the patients was procured by the facility. All patients were registered in a database created by the PRIME team and received a ‘smile card’, which is similar to an immunisation card and records the details of the patient and treatment provided. The PRIME team played a key role in facilitation of most of the activities mentioned above, especially related to identification of patients, organisation of outreach clinics and setting up the drug procurement and information systems. Psychotropic drugs mentioned in the essential drug list of Madhya Pradesh are now available in Bilkisgunj community health centres. These drugs are chlorpromazine, diazepam, alprazolam, amitriptyline, haloperidol, fluoxetine and vitamin B_1_ (thiamine). The monthly HMIS report on mental health indicators was regularly submitted by this facility from January 2014 onwards.

Despite these positive developments, the participation of general facility staff in the specialist clinics was minimal. The medical officers and paramedical staff (nurses) did not sit with the DMHP team during these clinics, citing other responsibilities and work commitments. Despite the clinics, very few people with priority disorders were identified at the facility by medical officers (the majority of patients being referred by the front-line workers) and even if they were identified they were only referred to specialist clinics and there was no initiation of either pharmacological management or psychosocial interventions. Users and carers expressed positive opinions regarding the specialist clinics as the services were now available closer to their villages compared with the district hospital in Sehore, but they also mentioned that the services should be available every day and not just during these specialist clinics. This would improve the accessibility of services as it is sometimes difficult for people to come on a particular day because of other commitments. Many users were lost to follow-up because of this barrier. They also expressed the need to be routinely treated in the out-patient department of the facility by medical officers as the services would be less stigmatising. It was observed that patients with psychosis who were earlier identified in the community were not able to attend the specialist clinics as there was no one to accompany them to the clinic. To mitigate this, some carers suggested that specialist clinics be conducted at the village level, at least for psychosis.

## Discussion

This paper describes the development and piloting of a comprehensive MHCP aimed at integrating the delivery of evidence-based interventions for three priority mental disorders (depression, psychosis and alcohol use disorders) in routine primary healthcare in a district in India. The emerging MHCP has seven packages that can be broadly divided into enabling and service delivery packages. The enabling packages essentially focus on establishing the foundation for facilitating the service delivery packages. The key lessons from the piloting of selected packages were that: (a) mental health service delivery can be strengthened only with strong facilitation by an external resource team such as the PRIME team in our case; (b) an additional human resource in the form of a case manager is essential to establish true collaborative models of care; (c) enabling packages need to be installed as a foundation prior to the implementation of service delivery packages; and (d) the focus of the MHCP from three priority disorders needs to be broadened to include patients with medically unexplained somatic presentations.

### Role of the PRIME in the MHCP implementation

Our initial plan prior to beginning this work had envisaged that most of the community- and facility-based activities would be conducted by existing human resources in the healthcare systems, namely the medical officers, paramedical staff in facilities and front-line workers in the community. The role of the PRIME and DMHP team would be restricted to capacity building of the staff and evaluation of outcomes. This is similar to the ‘training primary care staff’ model in the Bower & Gilbody framework of mental healthcare in primary care.^15^ We found that this design resulted in very poor performance, which is consistent with the global evidence.^16,17^ It was decided that the PRIME team would play a more active role in ‘facilitation’ of various programme activities and mobilised the DMHP team to conduct specialist clinics in the facility, thus establishing a ‘consultation–liaison’ model.^15^ In practice, however, it was observed that the PRIME team members had to play a very significant role in identification of patients in the community, provision of basic psychosocial counselling during the specialist clinics and in the activities related to the procurement and supply chain management and the HMIS components of the programme management package. Pharmacological treatment was entirely handled by the DMHP psychiatrist and the participation of medical officers in mental health service delivery was minimal. This was not integration of mental health in primary care in its true sense, but rather a ‘replacement’ model of integration that is both resource intensive and less effective for improving access and equity.^15^

### Mental health case managers: a new human resource

This challenge could potentially be addressed by establishing a collaborative stepped care model of care involving recruitment of an additional human resource in the form of a mental health case manager at the facility level. There is a strong evidence base supporting the effectiveness of collaborative stepped care models for treatment of mental disorders and the most crucial element in establishing this type of service delivery model is the case manager.^18^ Importantly, this evidence has also influenced the design of India's new DMHP that calls for the appointment of new mental health dedicated workers at the level of primary health centres.^6^ While awaiting the implementation of this new directive, the PRIME team used its resources to facilitate the recruitment of new mental health case managers to perform a range of case management tasks such as screening patients for depression, maternal depression and alcohol use disorders, providing psychosocial interventions and coordinating care with the medical officer. The case manager will establish a ‘mental health corner’ within the facility that will essentially be a dedicated space for all mental health interventions within the facility. At the time of preparation of this paper, the recruitment of six mental health case managers has been completed. They will now be trained and the collaborative care model will be delivered in the community health centres.

### Systems thinking for MHCP implementation

A key strength of this MHCP is the integration of the health system strengthening packages within the clinical delivery packages. De Savigny & Adam^19^ have made a strong case for systems thinking and have mentioned that, in unmapped and misunderstood systems, even the very simplest interventions often fail to achieve their goals. This is not necessarily because of any inherent flaw in the intervention itself but rather because of the often unpredictable behaviour of the system around it.^19^

Global mental health experts have emphasised the need for community mental health teams and their ability to carry out a broad array of tasks such as identification, referrals, elementary counselling, family support and psychosocial interventions, if they are provided adequate training and periodic monitoring and support.^20,21^ The role of psychiatrists also needs to undergo a fundamental paradigm shift and it is proposed that they should now play a major role in new core tasks such as designing mental healthcare programmes that can be delivered by these community mental health teams, capacity building and providing supportive supervision to members of these team.^22^

The PRIME India MHCP is in alignment with this zeitgeist in global mental health and it also makes another specific contribution by moving the focus from only clinical interventions (service delivery packages) to necessitating systems strengthening (enabling packages) for effective delivery of interventions.

One of the major limitations in the pilot implementation of the MHCP was our inability to implement all the packages of care mentioned in the original MHCP. In particular, the recovery package was not implemented as there was a lack of human resource to follow-up the patients, ensure adherence management and facilitate community-based rehabilitation. Maternal mental health interventions were provided to only six mothers and further efforts need to be made to screen mothers in the antenatal and postnatal period and provide psychosocial interventions. In the next phase though these interventions will be fully integrated into the MHCP. The implementation phase will end with definitive evaluation of the delivery of care packages^9^ and a final MHCP will be created based on these findings that will then be scaled up in the entire district, and subsequently in the state of Madhya Pradesh. A key to enabling this process is the role of the PRIME team in providing technical assistance to support, guide, evaluate and facilitate the implementation of the MHCP, particularly at this critical early stage of transforming the health system to integrate mental healthcare. In the long run, however, the government's own DMHP team is expected to make a transition from solely providing clinical services to playing the role of the PRIME team as well.

## References

[1] Institute for HealthMetrics andEvaluation (IHME) India: Global Burden of Disease Study. IHME, 2010 (http://www.healthdata.org/sites/default/files/files/country_profiles/GBD/ihme_gbd_country_report_india.pdf/).

[2] Patel V Araya R Chatterjee S Chisholm D Cohen A De Silva M Treatment and prevention of mental disorders in low-income and middle-income countries. Lancet 2007; 370: 991–1005. 17804058 10.1016/S0140-6736(07)61240-9

[3] Murthy RS Mental health initiatives in India (1947-2010). Natl Med J India 2011; 24: 98–107. 21668056

[4] Goel DS Why mental health services in low- and middle-income countries are underresourced, under-performing: an Indian perspective. Natl Med J India 2011; 24: 94–7. 21668055

[5] Ministry of Health & Family Welfare (MOHFW) The Mental Health Care Bill. MOHFW, 2013 (http://www.prsindia.org/billtrack/the-mental-healthcare-bill-2013-2864).

[6] Ministry of Health & Family Welfare (MOHFW) XIIth Plan District Mental Health Programme. MOHFW, 2012 (https://mhpolicy.files.wordpress.com/2012/07/final-dmhp-design-xii-plan2.pdf).

[7] Lund C Tomlinson M De Silva M Fekadu A Shidhaye R Jordans M PRIME: a programme to reduce the treatment gap for mental disorders in five low- and middle-income countries. PLoS Med 2012; 9: e1001359. 23300387 10.1371/journal.pmed.1001359 PMC3531506

[8] Craig P Dieppe P Macintyre S Michie S Nazareth I Petticrew M Developing and evaluating complex interventions: the new Medical Research Council guidance. BMJ 2008; 337: a1655. 18824488 10.1136/bmj.a1655 PMC2769032

[9] De Silva MJ Rathod SD Hanlon C Breuer E Chisholm D Fekadu A Evaluation of district mental healthcare plans: the PRIME consortium methodology. Br J Psychiatry 2015, in press (doi: 10.1192/bjp.bp.114.153858). 10.1192/bjp.bp.114.153858 PMC4698558 26447175

[10] Rahman A Surkan PJ Cayetano CE Rwagatare P Dickson KE Grand challenges: integrating maternal mental health into maternal and child health programmes. PLoS Med 2013; 10: e1001442. 23667345 10.1371/journal.pmed.1001442 PMC3646722

[11] Hanlon C Luitel NP Kathree T Murhar V Shrivasta S Medhin G Challenges and opportunities for implementing integrated mental health care: a district level situation analysis from five low- and middle-income countries. PLoS One 2014; 9: e88437. 24558389 10.1371/journal.pone.0088437 PMC3928234

[12] Vogel I Review of the Use of ‘Theory of Change’. Department for International Development (DFID), 2012.

[13] Breuer E De Silva MJ Shidaye R Petersen I Nakku J Jordans MJD Planning and evaluating mental health services in low- and middle-income countries using theory of change. Br J Psychiatry 2015, in press (doi: 10.1192/bjp.bp.114.153841). 10.1192/bjp.bp.114.153841 PMC4698557 26447178

[14] World Health Organization Mental Health Gap Action Programme (mhGAP): Scaling Up Care for Mental, Neurological and Substance Abuse Disorders. WHO, 2008. 26290926

[15] Bower P Gilbody S Managing common mental health disorders in primary care: conceptual models and evidence base. BMJ 2005; 330: 839–42. 15817554 10.1136/bmj.330.7495.839 PMC556082

[16] Jenkins R Othieno C Okeyo S Kaseje D Aruwa J Oyugi H Short structured general mental health in service training programme in Kenya improves patient health and social outcomes but not detection of mental health problems - a pragmatic cluster randomised controlled trial. Int J Ment Health Syst 2013; 7: 25. 24188964 10.1186/1752-4458-7-25 PMC4174904

[17] Goncalves DA Fortes S Campos M Ballester D Portugal FB Tófoli LF Evaluation of a mental health training intervention for multidisciplinary teams in primary care in Brazil: a pre- and posttest study. Gen Hosp Psychiatry 2013; 35: 304–8. 23521815 10.1016/j.genhosppsych.2013.01.003

[18] Patel V Weiss HA Chowdhary N Naik S Pednekar S Chatterjee S Effectiveness of an intervention led by lay health counsellors for depressive and anxiety disorders in primary care in Goa, India (MANAS): a cluster randomised controlled trial. Lancet 2010; 376: 2086–95. 21159375 10.1016/S0140-6736(10)61508-5 PMC4964905

[19] De Savigny D Adam T Systems Thinking for Health Systems Strengthening. Alliance for Health Policy and Systems Research. WHO, 2009.

[20] Thornicroft G Tansella M Are community mental health services relevant in low- and middle-income countries? Epidemiol Psychiatr Sci 2014; 23: 115–8. 24642109 10.1017/S2045796014000067 PMC6998198

[21] Thara R John S Chatterjee S Community mental health teams in low- and middle-income countries. Epidemiol Psychiatr Sci 2014; 23: 119–22. 24513439 10.1017/S2045796014000079 PMC6998190

[22] Kigozi F Ssebunnya J The multiplier role of psychiatrists in low income settings. Epidemiol Psychiatr Sci 2014; 23: 123–7. 24576614 10.1017/S2045796014000080 PMC6998189

